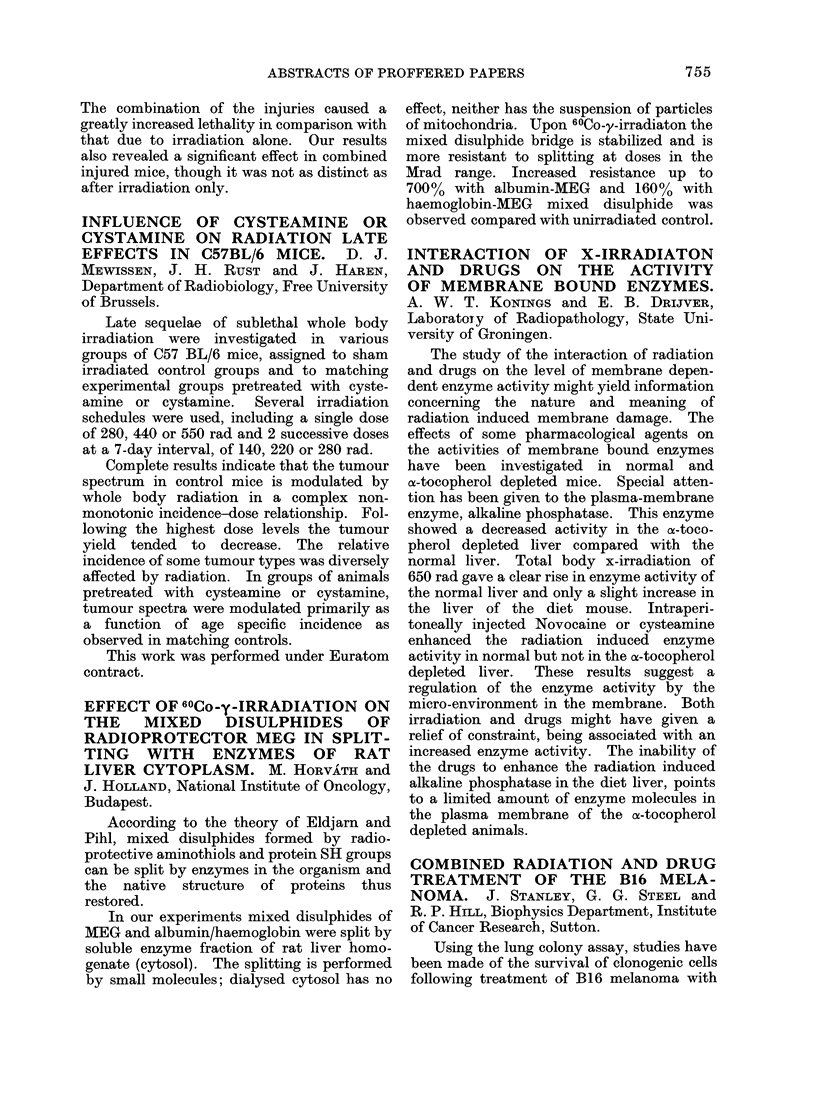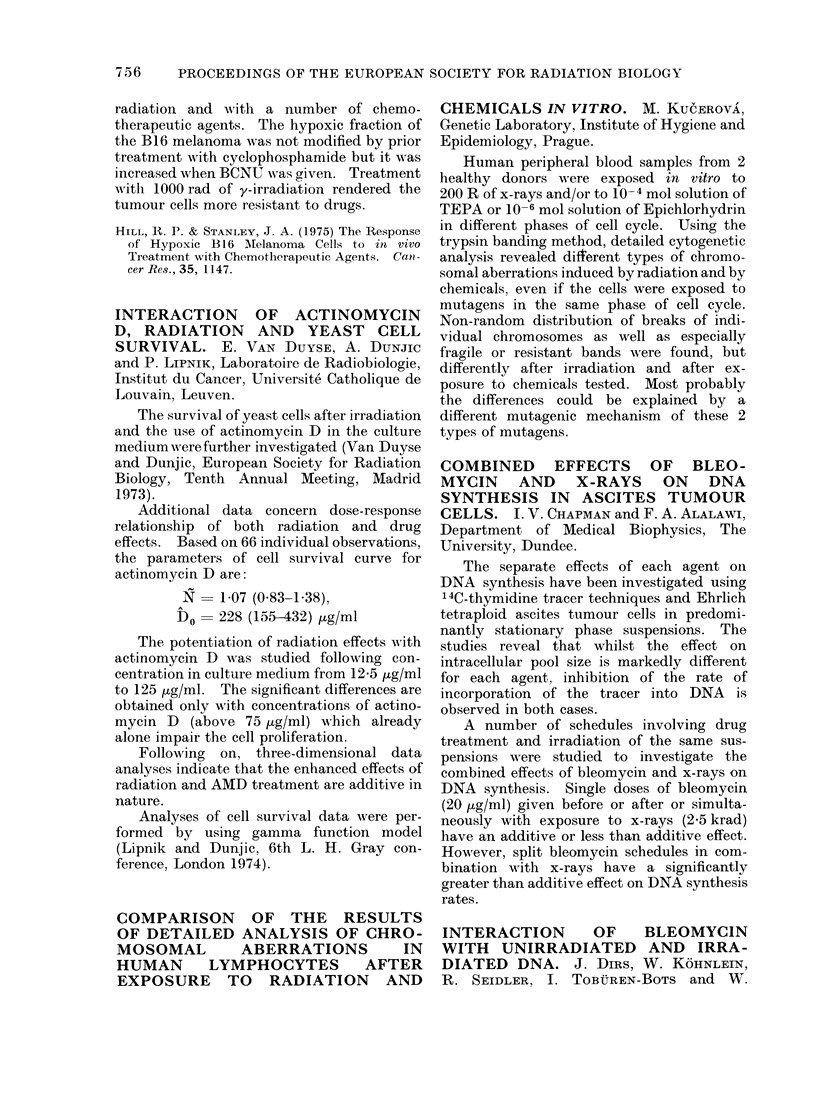# Proceedings: Combined radiation and drug-treatment of the B16 melanoma.

**DOI:** 10.1038/bjc.1975.303

**Published:** 1975-12

**Authors:** J. Stanley, G. G. Steel, R. P. Hill


**COMBINED RADIATION AND DRUG TREATMENT OF THE B16 MELANOMA.** J. Stanley, G. G. Steel and R. P. Hill, Biophysics Department, Institute of Cancer Research, Sutton.

Using the lung colony assay, studies have been made of the survival of clonogenic cells following treatment of B16 melanoma with radiation and with a number of chemotherapeutic agents. The hypoxic fraction of the B16 melanoma was not modified by prior treatment with cyclophosphamide but it was increased when BCNU was given. Treatment with 1000 rad of $\gamma$-irradiation rendered the tumour cells more resistant to drugs.

HILL, R. P. & STANLEY, J. A. (1975) The Response of Hypoxic B16 Melanoma Cells to *in vivo* Treatment with Chemotherapeutic Agents. *Cancer Res.*, **35**, 1147.